# 

**Published:** 1819-04

**Authors:** 


### MONTHLY CATALOGUE OF MEDICAL BOOKS.

An Essay on the Diseases of the Excretory Parts of the Lachrymal Organs; by William MacKenzie. 8vo. 4s. 6d.

Aphorisms, illustrating natural and difficult Cases of Labour, Uterine Hæmorrhage, and Puerperal Peritonitis; adapted to the use of Students; by Andrew Blake, M.D. 8vo. 3s. 6d.

The Quarterly Journal of Foreign Medicine and Surgery, and of the Sciences connected with them. No. II. for February 1819. 8vo. 3s. 6d.

Observations on Contagion, as it relates to the Plague and other Epidemic Diseases, and refers to the Regulations of Quarantine; by a Physician. 8vo. 2s. 6d.

An Essay on the Diagnosis between Erysipelas, Phlegmon, and Erythema; with an Appendix, touching the probable Nature of Puerperal Fever; by G. H. Weatherhead, M.D. &c. 8vo.

Further Observations on the Internal Use of the Hydro-cyanic (Prussic) Acid in Pulmonary Complaints, Chronic Catarrhs, Spasmodic Coughs, Asthma, Hooping Cough, and some other Diseases; with full Directions for the Preparation and Administration of that Medicine. By A. B. Granville, M.D. F.R.S. F.L.S. M.R.I. &c. 8vo. 4s. 6d.

A Letter to the Right Hon. F. Robinson, M.P. on the Plague and Contagion, with Reference to the Quarantine Laws; from A. B. Granville, M.D. F.R.S. F.L.S. M.R.I. &c. 8vo. 4s.

Observations on the Inflammatory Endemic incidental to Strangers in the West-Indies from temperate Climates, commonly called the Yellow Fever; as this Disease occurred to the Writer during a public Service of twenty Years in a majority of the West-India Colonies. By Nodes Dickinson, of the Royal College of Surgeons, &c. 8vo. 8s.

An Inquiry into the Effects produced on the Brain, Lungs, and other Viscera, and on the Nervous System, by Diseases of the Liver. By Thomas Mills, M.D. 8vo. 3s. 6d.

Additional Experiments on the Arteries of Warm-Blooded Animals. By C. H. Parry, M.D. F.R.S. 8vo. 12s.

Remarks on the Treatment of some of the most prevalent Varieties of Inflammation of the Eye; with Cases. By Thomas Whately. 8vo. 3s.

Observations on the Prevention and Treatment of the Epidemic Fever at present prevailing in this Metropolis, and most Parts of the United Kingdom. By Henry Clutterbuck, M.D. 8vo. 8s.

An Essay on Warm, Cold, and Vapour Bathing; with Practical Observations on Sea-Bathing, Diseases of the Skin, Bilious, Liver Complaints, and Dropsy. By Sir Arthur Clarke, M.D. &c. 12mo. 4s. 6d.

